# ERMO3/MVP1/GOLD36 Is Involved in a Cell Type-Specific Mechanism for Maintaining ER Morphology in *Arabidopsis thaliana*

**DOI:** 10.1371/journal.pone.0049103

**Published:** 2012-11-14

**Authors:** Ryohei Thomas Nakano, Ryo Matsushima, Atsushi J. Nagano, Yoichiro Fukao, Masayuki Fujiwara, Maki Kondo, Mikio Nishimura, Ikuko Hara-Nishimura

**Affiliations:** 1 Department of Botany, Graduate School of Science, Kyoto University, Kyoto, Japan; 2 Graduate School of Biological Sciences, Nara Institute of Science and Technology, Ikoma, Japan; 3 Department of Cell Biology, National Institute for Basic Biology, Okazaki, Japan; Peking University Health Science Center, China

## Abstract

The endoplasmic reticulum (ER) has a unique, network-like morphology. The ER structures are composed of tubules, cisternae, and three-way junctions. This morphology is highly conserved among eukaryotes, but the molecular mechanism that maintains ER morphology has not yet been elucidated. In addition, certain *Brassicaceae* plants develop a unique ER-derived organelle called the ER body. This organelle accumulates large amounts of PYK10, a β-glucosidase, but its physiological functions are still obscure. We aimed to identify a novel factor required for maintaining the morphology of the ER, including ER bodies, and employed a forward-genetic approach using transgenic *Arabidopsis thaliana* (GFP-h) with fluorescently-labeled ER. We isolated and investigated a mutant (designated *endoplasmic reticulum morphology3*, *ermo3*) with huge aggregates and abnormal punctate structures of ER. *ERMO3* encodes a GDSL-lipase/esterase family protein, also known as MVP1. Here, we showed that, although ERMO3/MVP1/GOLD36 was expressed ubiquitously, the morphological defects of *ermo3* were specifically seen in a certain type of cells where ER bodies developed. Coimmunoprecipitation analysis combined with mass spectrometry revealed that ERMO3/MVP1/GOLD36 interacts with the PYK10 complex, a huge protein complex that is thought to be important for ER body-related defense systems. We also found that the depletion of transcription factor NAI1, a master regulator for ER body formation, suppressed the formation of ER-aggregates in *ermo3* cells, suggesting that NAI1 expression plays an important role in the abnormal aggregation of ER. Our results suggest that ERMO3/MVP1/GOLD36 is required for preventing ER and other organelles from abnormal aggregation and for maintaining proper ER morphology in a coordinated manner with NAI1.

## Introduction

The endoplasmic reticulum (ER) forms a highly complicated meshwork of structures that consist of ER tubules and ER cisternae. This meshwork actively changes its structure and moves around the cell through the process of cytoplasmic streaming (ER streaming; [1]). The molecular mechanisms underlying the formation and maintenance of ER morphology has been investigated in this decade using animal and yeast cells [2], [3]. Reticulon family proteins, the most well-known ER structural proteins, are membrane proteins that mechanically bend the ER membrane at its hairpin-like, hydrophobic segments [4]–[6]. Similar roles are played by Sey1p in yeast cells and Atlastin family proteins in animal cells. Interestingly, these proteins also possess a GTPase domain, which is important for their role in maintaining ER morphology [7], [8]. In contrast to the high degree of curvature of membranes in ER tubules, the membranes in ER cisternae are arranged in flat planes, a structure that is maintained in mammalian cells by another membrane protein, Climp63 [9]. Most of these proteins, including reticulons and atlastins, are conserved in plant cells and may be involved in regulating ER morphology [10]–[16].

In plant cells, it is known that actin filaments are required for the movement and morphology of the ER meshwork [17]–[20]. Recently, it has been shown that impaired ER streaming, due to the loss of certain Myosin XI proteins (IX-K, MYA-1, and MYA-2), caused abnormal organization of actin filaments, suggesting that the organization of ER and actin filaments is mutually regulated [1]. GNOM-LIKE1/ERMO1 and SEC24A/ERMO2 of *Arabidopsis* have also been shown to be involved in maintaining ER morphology by transporting some unknown key factors [20], [21].

Despite intense studies to understand the static mechanisms required to maintain ER morphology that focused on a single or a few proteins, questions still remain concerning the regulation of tubule and cisterna formation and localization, the mechanisms underlying dynamic structural changes, and the biological significance of ER morphology (reviewed by [14]).

*Arabidopsis* and other *Brassicaceae* plants contain a unique, ER-derived structure called the ER body. The ER body is statically continuous with the ER and is clearly visualized using ER-localized green fluorescent protein (GFP) [22], [23]. ER bodies develop in epidermal cells of seedlings and roots but rarely in mature aerial tissues. Combined with the observation that ER bodies are induced both locally and systemically by wounding and methyl jasmonate (MeJA) treatment [23], [24], ER bodies are thought to be involved in plant defenses [25]. PYK10, the major component of ER bodies, is a member of the β-glucosidase family. Previous studies have shown that PYK10 forms a large protein complex of up to 70 µm [26]. One of the PYK10 complex components is PYK10-BINDING PROTEIN1 (PBP1), a member of jacalin-related lectins (JALs) [26] and PBP1 activates PYK10 without any cofactors [27], suggesting that the formation of the PYK10 complex facilitates activation of PYK10 enzymatic activities. Presuming that PYK10 enzymatic activities are important for the physiological functions of ER bodies, formation of PYK10 complex may play an important role in the plant defense activity of the ER body system.

To understand how these ER structures, including ER bodies, are formed, and to identify a novel factor that is required for maintaining ER morphology, we employed a forward-genetic approach using transgenic *Arabidopsis* (GFP-h) that expresses GFP fused N-terminally with signal peptide of pumpkin 2S albumin and C-terminally with an ER-retention signal sequence, His-Asp-Glu-Leu (SP-GFP-HDEL). We isolated an *endoplasmic reticulum morphology3* (*ermo3*) mutant that contains ER arranged in a large aggregate. We found that the loss of ERMO3 caused the ER to aggregate where the transcription factor NAI1 was expressed.

## Results

### The *ermo3-1* mutant develops huge aggregates of ER

**Figure 1:**
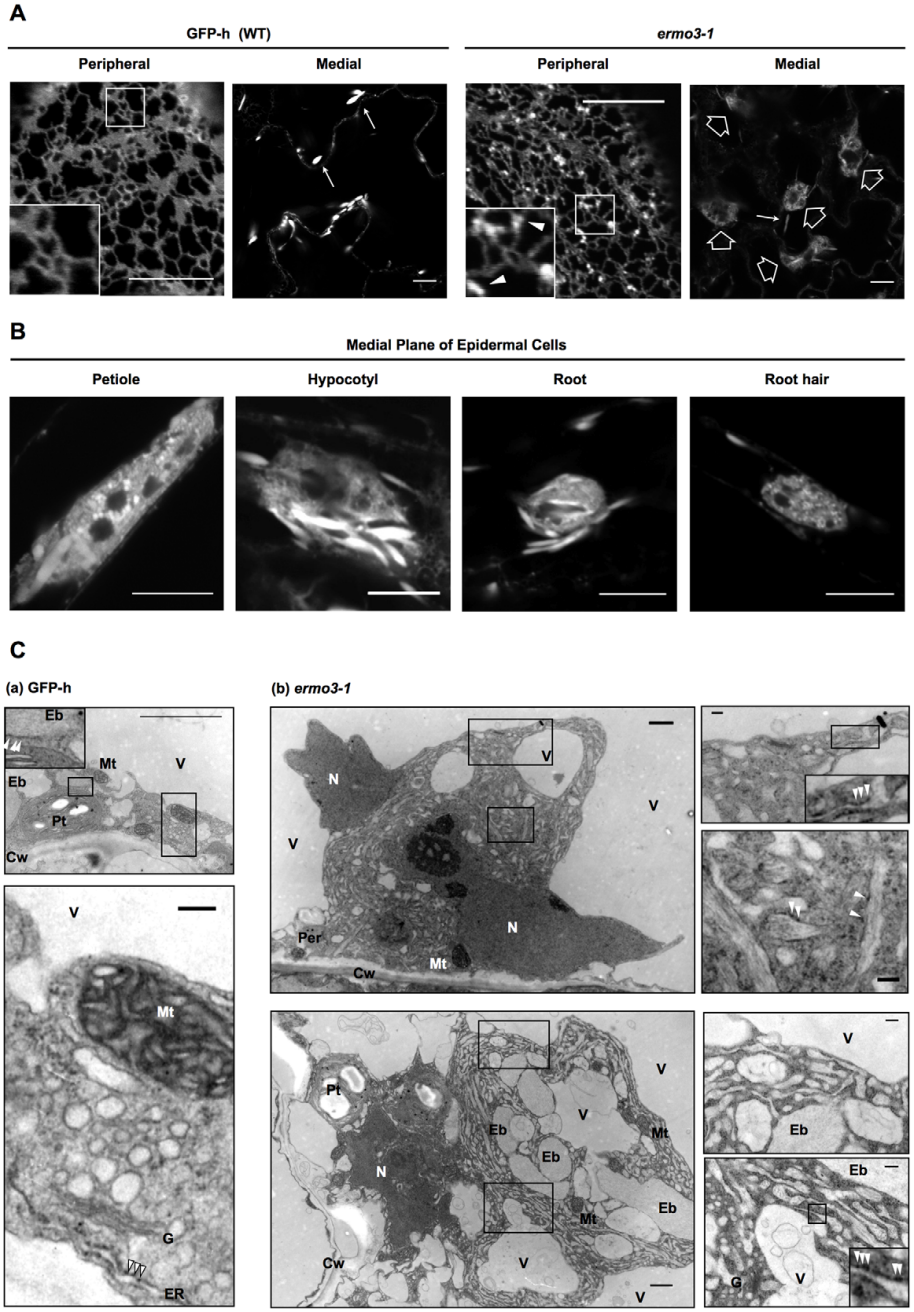
*ermo3-1* developed a large aggregate composed of ER-derived aberrant structures and various organelles. (**A**) Transgenic *Arabidopsis* (GFP-h) expressing SP-GFP-HDEL was observed under a confocal microscope (left panels, WT). *ermo3-1* was isolated from chemically mutagenized GFP-h seeds and subjected to confocal microscopy (right panels). In GFP-h cells, the typical ER network (peripheral, inset) and ER bodies (medial, arrows) were seen. In *ermo3-1* cells, punctate structures (peripheral, arrowheads in inset) and huge aggregates (medial, open arrows) were seen in addition to the typical network and ER bodies. Bars, 10 µm. (**B**) Epidermal cells of petioles, hypocotyls, roots, and root hairs from 7-day-old *ermo3-1* developed ER-aggregates, as seen in cotyledons. Bars, 10 µm. (**C**) Transmission electron micrographs of GFP-h (**a**) and *ermo3-1* (**b**) cells. ER was usually seen as a thin structure in the periphery surrounded by ribosomes (arrowheads). ER bodies were recognized as large and relatively electron-dense compartments (inset). The *ermo3-1* aggregates appeared as huge structures consisting of various structures. Abnormal swollen structures were occasionally surrounded by ribosomes (arrowheads) and connected with ER cisternae (insets). Eb, ER body; V, vacuole; G, Golgi body; N, nucleus; Mt, mitochondrion; Per, peroxisome; Cw, cell wall; Pt, plastid. Bars, 1 µm (upper panel of [**a**] and left column of [**b**]) and 200 nm (lower panel of [**a**] and right column of [**b**]).

We isolated a recessive mutant that had defective ER morphology from chemically mutagenized GFP-h and designated it the *endoplasmic reticulum morphology3-1* (*ermo3-1*) mutant. This mutant developed a huge aggregate of ER and a number of punctate structures on the peripheral meshwork of cotyledon cells (Figure 1A). This aggregation of ER was also seen in petioles, hypocotyls, roots, and root hairs (Figure 1B). Electron microscopy revealed that the ER-aggregate in an *ermo3-1* cell comprised a huge aggregate of cytoplasm. In wild type cells, most organelles are distributed in a thin layer between plasma membrane and vacuolar membrane (Figure 1Ca). Mitochondria and peroxisomes were recognized as circular structures and plastids were found as large compartments with internal membranes and starch granules. Vacuoles were pushing cytoplasm towards plasma membrane and vacuolar lumens showed relatively less electron density. ER bodies, as described previously, were gray-colored and surrounded by ribosomes (arrowheads in an inset). In the enlarged view (bottom panel), ER and Golgi bodies were closely contacting each other. ER had a number of ribosomes on its cytosolic surface (arrowheads). In *ermo3-1* cells, on the other hand, these organelles were engulfed into the aggregate of cytoplasm (Figure 1Cb). It contained various organelles such as vacuoles, ER bodies, Golgi bodies, mitochondria, peroxisomes, nuclei, and plastids (left column). This result was consistent with results obtained by confocal microscopic analysis (Figure S1). In addition to the clearly identifiable organelles, the cytoplasm was also filled with abnormal structures. Enlarged views of these structures are shown in the right column of Figure 1Cb. These abnormal structures had ca. 100 nm–200 nm thickness, which was thicker than typical ER cisternae (less than 100 nm). We found them surrounded by ribosomes (indicated by arrowheads) and occasionally connected to typical ER cisternae (insets), indicating that these abnormal structures were derived from ER. These results show that the isolated *ermo3-1* mutant had morphological defects in the ER and aberrant intracellular distribution of not only ER but also most organelles.

**Figure 2:**
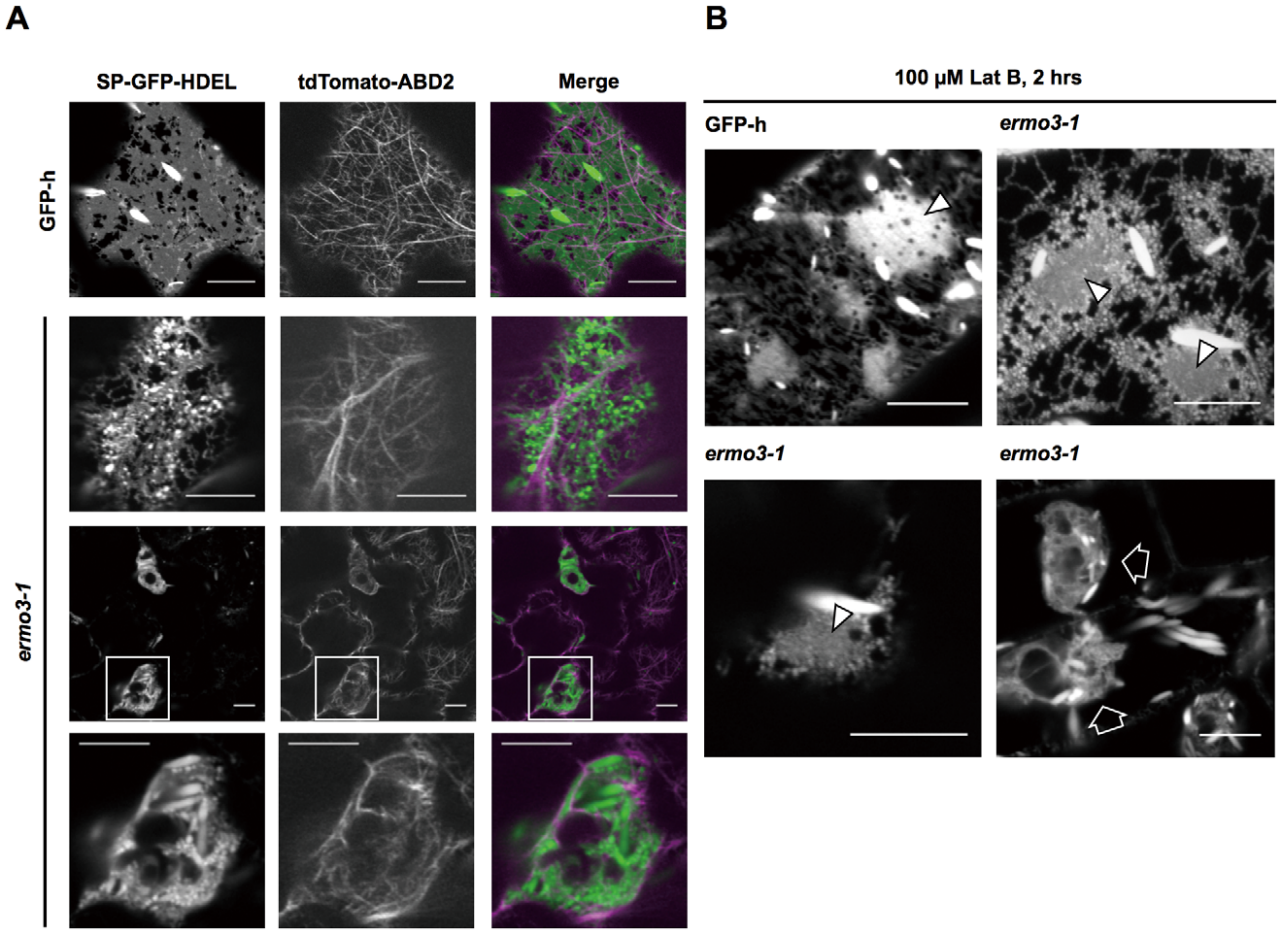
Actin filaments were properly organized in *ermo3-1* cells. (**A**) Confocal images obtained from T1 plants of *ermo3-1* transformed by *tdTomato-ABD2*. Panels in the middle row are enlarged views of top panels. The aggregates were surrounded by organized actin filaments. Bottom row images show that the peripheral actin network was properly organized. Bars, 10 µm. (**B**) Seedlings of GFP-h and *ermo3-1* were treated with 100 µM of Latrunculin B (Lat B), an actin inhibitor, for 2 hours. Green arrowheads indicate Lat B-induced aggregates, which were derived from collapsed ER networks. In the presence of Lat B, *ermo3* aggregates (open arrows) were still similar to those of non-treated cells. Bars, 10 µm.

To determine whether *ermo3-1* had defects in the organization of actin filaments, we expressed tdTomato-ABD2, a fluorescent actin marker protein, in *ermo3-1* and GFP-h plants (Figure 2A). In GFP-h, typical actin networks, which are composed of a number of thin filaments and several thick bundles, were seen (Figure 2A, GFP-h). Similarly, *ermo3-1* also developed typical actin networks, and the ER strands were aligned on thick actin bundles. The aggregate in *ermo3-1* cells was caged and penetrated with actin filaments (Figure 2A, *ermo3-1*). Disruption of these “actin cages” by an actin polymerization inhibitor (latrunculin B, Lat B) did not suppress the formation of aggregates (Figure 2B, open arrows). This inhibitor treatment induced the collapse of the ER meshwork, and the formation of abnormal ER structures as was also seen in GFP-h (Figure 2B, arrowheads), but the inhibitor not affect the nature of ER-aggregates in *ermo3-1* cells. Our findings are summarized as follows: 1) the actin network was properly organized in *ermo3-1* cells, 2) ER interacted with actin filaments in *ermo3-1* cells and in wild-type cells in a similar manner, and 3) the formation of ER-aggregates in *ermo3-1* cells was independent of actin cages. These findings suggest that *ermo3-1* had defects at either downstream of the actin-ER interaction or in another molecular mechanism independent of actin filaments.

### *ERMO3* is identical to *MODIFIED VACUOLE PHENOTYPE1* encoding a GDSL-lipase/esterase family protein

**Figure 3:**
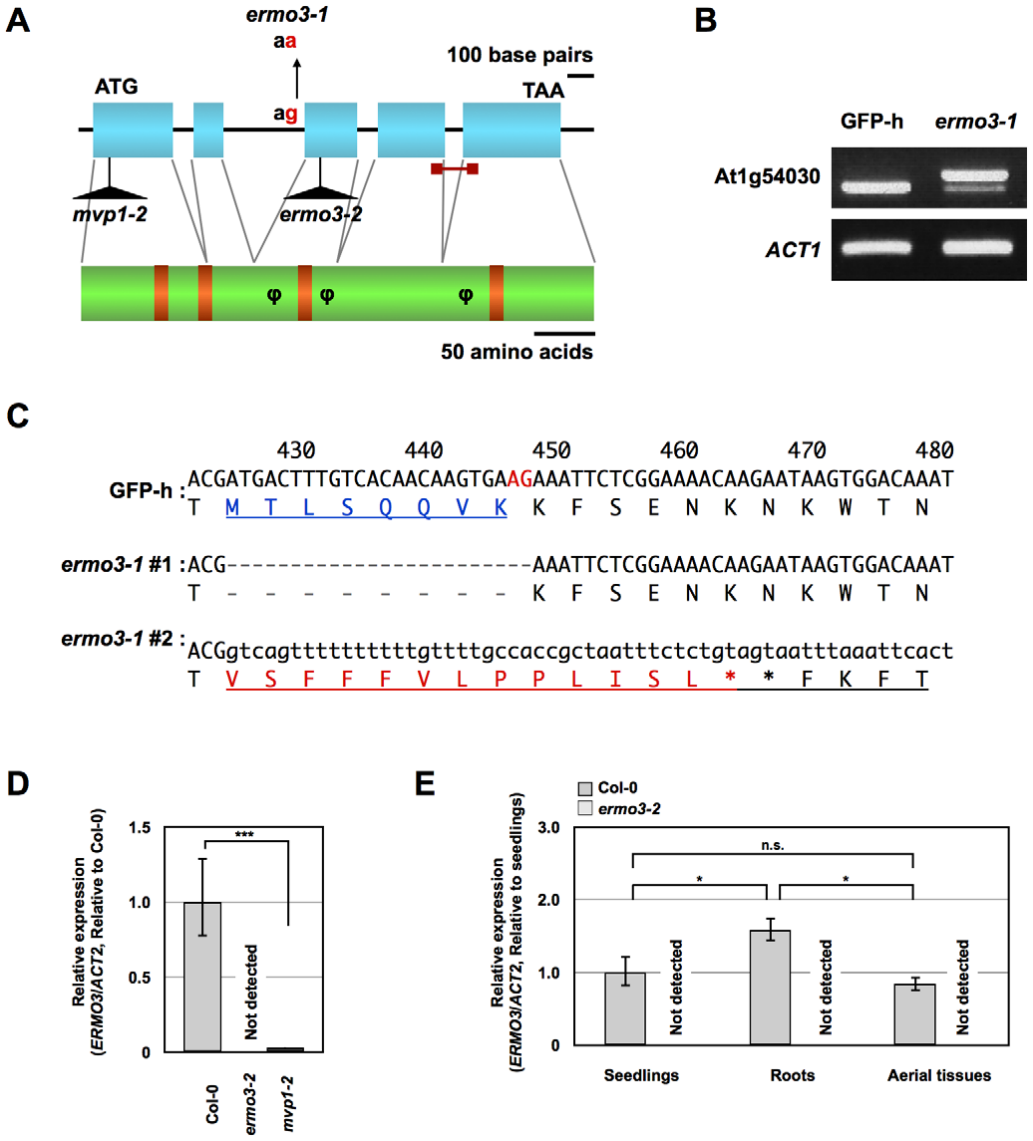
Characterization of *ERMO3* gene. (**A**) Gene model and protein structure of At1g54030. Base-pair substitution found in *ermo3-1* and T-DNA insertion sites of *ermo3*-2 (SALK_135215) and *mvp1-2* (SALK_030621) are shown. Blue boxes and black lines indicate exons and introns, respectively. Conserved domains for catalytic triad and oxyanion holes are indicated by orange boxes. Φ indicates a putative glycosylation site. (**B**) RT-PCR using total RNA extracted from 7-day-old seedlings of GFP-h and *ermo3-1* amplified full-length ERMO3. *ermo3-1* transcribed two distinct mRNAs. *ACT1* was used for the internal control. (**C**) Sequencing results of mRNA transcribed in GFP-h and *ermo3-1* around the substitution site. Predicted protein sequences are also shown. Numbers above sequences indicate positions in CDS. #1 and #2 of *ermo3-1* correspond to shorter and longer mRNAs shown in (**B**), respectively. (**D**) Quantitative real-time PCR (qPCR) using total RNA from 7-day-old seedlings of CS60000, *ermo3*-2, and *mvp1-2*. The amplified region is indicated by a red bar in (**A**). *ERMO3* expression in *ermo3-2* was not detected and little in *mvp1-2* (*** *P*<0.0001). (**E**) Total RNA from seedlings (7-day-old), roots (2-week-old), and aerial tissues (2-week-old) of GFP-h and *ermo3-2* were subjected to qPCR. *ERMO3* expression was detected in all three tissues of Col-0, while none was detected in *ermo3-2*. * *P*<0.01, ** *P*<0.001, *** *P*<0.0001.

We examined the *ermo3-1* genome and were able to identify a single base pair substitution at the At1g54030 locus (Figure 3A). In the *ermo3-1* genome, an adenine-guanine sequence at the splicing junction between the second intron and the third exon was substituted with an adenine-adenine sequence. This substitution caused a splicing defect in expression of this locus, as revealed by RT-PCR (Figure 3B). The two most prominently transcribed mRNAs in *ermo3-1* seedlings were sequenced (Figure 3C). The shorter mRNA (*ermo3-1* #1) was spliced using another adenine-guanine sequence (indicated by red characters in the wild-type nucleotide sequence), resulting in a 24 base-pair deletion. The protein translated from this mRNA has an eight-amino-acid deletion (indicated by blue characters in the wild-type protein sequence). On the other hand, the second intron was not spliced out in the longer mRNA (*ermo3-1* #2), resulting in the translation of a 13 amino acid insertion followed by a stop codon (indicated by red characters in the *ermo3-1* #2 protein sequence). We then isolated two T-DNA inserted lines (SALK_030621, also known as *mvp1-2*, and SALK_135215), and the expression levels of this locus were investigated by quantitative real-time PCR (qPCR; Figure 3D). The results showed that *mvp1-2* expressed little mRNA (3.0% of Col-0, with *P*-value of 0.000013), and SALK_135215 was an additional knock-out allele of this locus. Loss of mRNA expression in *ermo3-2* was also seen both in roots and aerial tissues (Figure 3E). We stably introduced the SP-GFP-HDEL protein into SALK_135215 and found that this line also contained ER-aggregates and ER-derived punctate structures, both of which were similar to those in *ermo3-1* cells (Figure S2A). We crossed *ermo3-1* with *mvp1-2*, and the resulting F1 individuals also contained ER-aggregates and the punctate structures (Figure S2B). Together, these results indicate that *ERMO3* is located on At1g54030 and that the morphological defects observed in *ermo3-1* was not specifically due to splicing defects of this locus.

*ERMO3* is the same gene as the recently reported *MODIFIED VACUOLE PHENOTYPE1* (*MVP1* [28]) and *GOLD36* [29], which encode a GDSL-lipase/esterase family protein. The *mvp1* and *gold36* mutants were identified by the aberrant morphology of vacuolar membrane (GFP-deltaTIP) and Golgi bodies (ST-GFP), respectively, which is consistent with our observation that various organelles including vacuoles and Golgi bodies were engulfed in the ER-aggregates in *ermo3*. The molecular mechanism by which these aggregates were formed remained unsolved in the report by Agee *et al* [28]. Marti *et al.* concluded that the export of ERMO3/MVP1/GOLD36 out of the ER was required for the maintenance of organelle organization [29].

### ERMO3/MVP1/GOLD36 is transported to vacuoles but its functioning sites might be between ER and Golgi bodies

To understand the molecular mechanism involved in maintaining ER morphology, we focused on the subcellular localization of ERMO3/MVP1/GOLD36. At first, this localization remained unclear. Marti *et al.* concluded that ERMO3/MVP1/GOLD36 was localized in the vacuole [29], while Agee *et al.* initially reported that this protein was localized at the ER, ER bodies, and tonoplast [28]. The results of Marti *et al.* were based on experiments using GOLD36-mRFP transgenic plants, while Agee *et al.* based their results on MVP1-GFP transient expression assay data. We used another fluorescent protein, tagRFP, in combination with our knock-out allele, *ermo3-2*, under the 35S constitutive-expression promoter (Figure S3A). ERMO3-tagRFP labeled the vacuolar lumen and the ER, but not aggregates, indicating that this protein functioned well enough to suppress the formation of ER-aggregates. To reveal if labeling of the ER was an artificial effect of overexpression, we treated ERMO3-tagRFP expressing plants with a ribosome inhibitor (cycloheximide, CHX). After treatment for 2 hours, entire signals disappeared from the ER while the vacuolar lumen still fluoresced (Figure S3A, right panel). This suggests that ERMO3-tagRFP was translated in the ER and transported to the vacuolar lumen, further supporting the conclusion of Marti *et al*.

ERMO3/MVP1/GOLD36 is a member of the GDSL-lipase/esterase family and appears to be a pseudoenzyme [28]. We generated a cDNA encoding ERMO3^G59S^ with an artificially introduced catalytic-center residue (Figure S3B). This protein was C-terminally fused with tagRFP and introduced into the *ermo3-1* mutant under control of the 35S promoter (Figure S3C). Confocal microscopy revealed that this fused protein was transported to the vacuole and complemented the *ermo3-1* mutant, suggesting that ERMO3/MVP1/GOLD36 can maintain ER morphology regardless of whether it has enzymatic activity.

**Figure 4:**
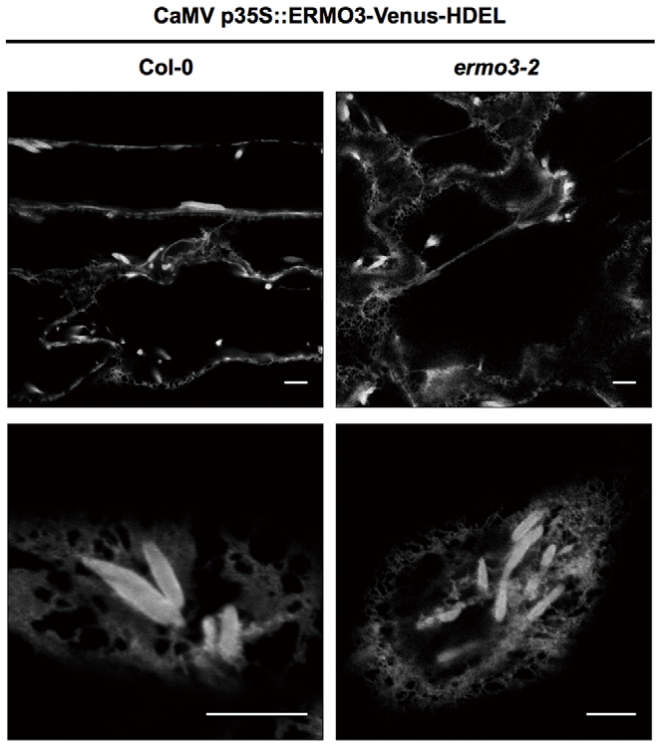
Cycling between ER and Golgi bodies was sufficient for ERMO3/MVP1/GOLD36 to suppress ER aggregation. ERMO3/MVP1/GOLD36 protein fused with Venus followed by the ER-retention signal sequence (His-Asp-Glu-Leu) in its C-terminus was stably expressed in Col-0 and in *ermo3*-2. In both lines, ERMO3-Venus-HDEL localized in the ER lumen and ER bodies. Notably, neither of these developed abnormal aggregates that were seen in *ermo3* cells.

We concluded that ERMO3/MVP1/GOLD36 was transported to the vacuole, but the morphological defects exhibited by this mutant occurred in the ER (see Figure 1C). Thus, we then examined the importance of vacuolar transport of ERMO3-tagRFP in the maintenance of ER morphology. To do this, we generated an *ERMO3-Venus-HDEL* construct. A protein that bears the HDEL sequence at the C-terminus will exit the ER, go to the Golgi bodies, and immediately return to the ER [30], [31], resulting in predominant localization at the ER. We introduced this construct into either *ermo3*-2 or Col-0 and observed the ER morphology through the fluorescence of ERMO3-Venus-HDEL (Figure 4). Unexpectedly, the ER morphology in the transformants was not defective, and no aggregated ER nor ER-derived punctate structures were found, indicating that ERMO3-Venus-HDEL was functional enough to maintain ER morphology. This suggests that, although native ERMO3/MVP1/GOLD36 seemed to be transported to the vacuole, its role in maintaining ER morphology was exerted between the ER and Golgi bodies. However, since ER-retention by HDEL is not completely robust, we could not exclude the possibility that a part of ERMO3-Venus-HDEL was transported to vacuole via Golgi bodies and this amount of transport was still enough for ERMO3/MVP1/GOLD36 to maintain ER morphology (discussed below).

### ERMO3/MVP1/GOLD36 interacts with PYK10 complex components

**Figure 5:**
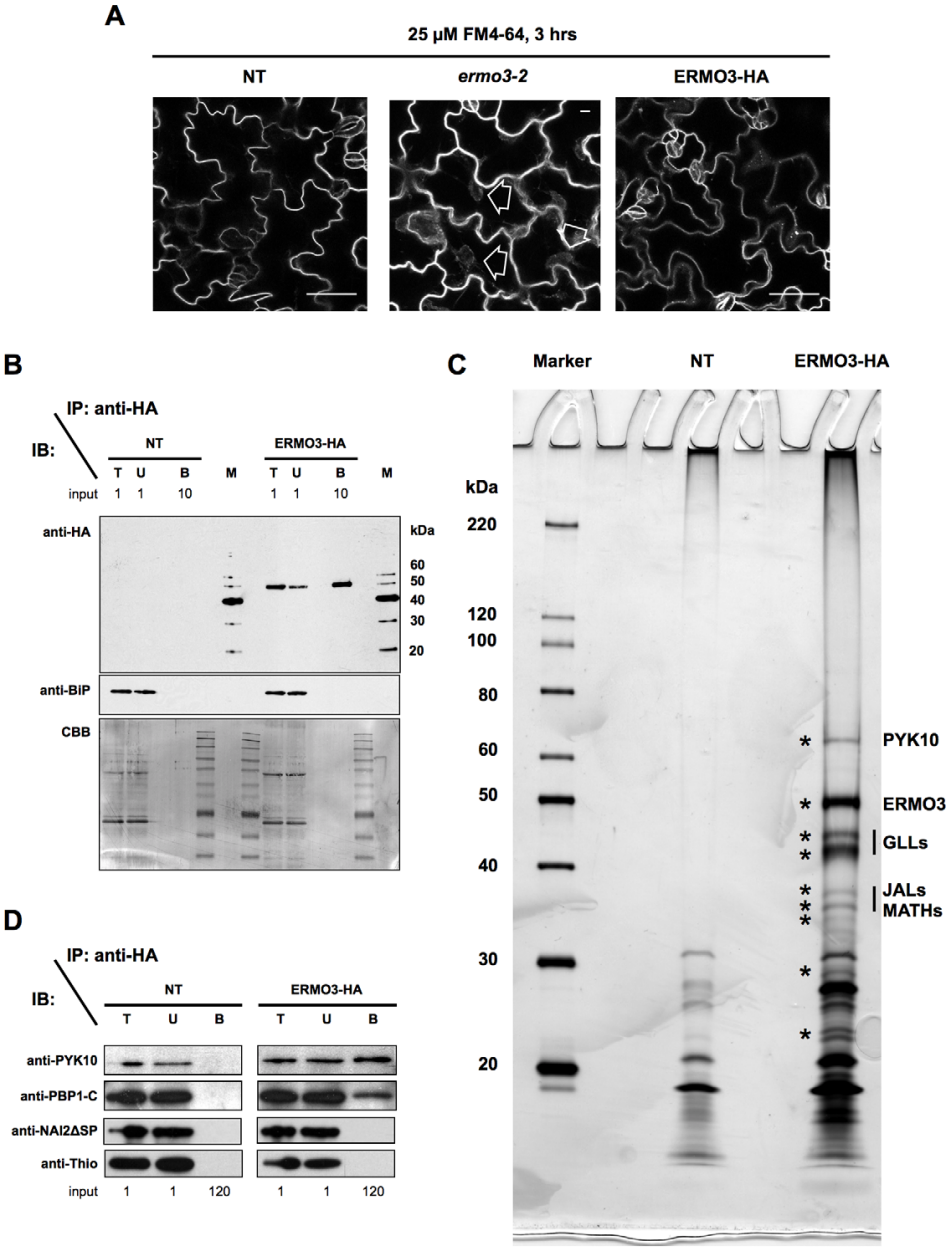
ERMO3/MVP1/GOLD36 interacts with PYK10 complex components. (**A**) Seven-day-old seedlings of NT (non-transgenic, identical to Col-0), *ermo3*-2, and ERMO3-HA were stained with 25 µM FM4-64 for 3 hours. ERMO3-HA did not develop any aggregates, unlike *ermo3*-2 (open arrows). (**B**) to (**D**) Seven-day-old seedlings of NT and ERMO3-HA were subjected to a co-immunoprecipitation (IP) assay using anti-HA antibodies. Total (T), unbound (U), and bound (B) fractions ([**B**] and [**D**]), or only the bound fraction (**C**) from NT and ERMO3-HA, were separated by SDS-PAGE followed by either immunoblot analysis (IB; top and middle panels in [**B**], and [**D**]), silver staining (**C**), or Coomassie Brilliant Blue (CBB) staining (bottom panel in [**B**]). Numbers shown in (**B**) and (**D**) indicate relative amounts of loaded fractions. Interacting proteins identified by mass spectrometry are indicated in (**C**). GLLs, GDSL lipase-like proteins; JALs, jacalin-related lectins; MATHs, meprin and TRAF homology [MATH] domain-containing proteins.

To reveal the molecular function of ERMO3/MVP1/GOLD36, we next focused on its interaction partners. We employed coimmunoprecipitation (Co-IP) analysis using a transgenic *Arabidopsis* line (designated ERMO3-HA) that stably expressed hemagglutinin- (HA-) tagged ERMO3/MVP1/GOLD36. We confirmed the expression of the tagged proteins in seedlings, which we primarily used to observe ER morphology. Furthermore, to obtain comprehensive insights into the interactome of ERMO3/MVP1/GOLD36, we performed mass spectrometric (MS) analysis after the Co-IP analysis. When the cells were stained with FM4-64 for 3 hours, ERMO3-HA cells, like the non-transgenic control (NT, identical to Col-0), did not contain obvious aggregates, while *ermo3*-2 cells had fluorescent aggregates, confirming that the ERMO3-HA protein was functional (Figure 5A). Immunoblot analyses using either the anti-HA or anti-BiP (ER-resident protein as a control) antibody, followed by CBB staining, confirmed that ERMO3-HA was efficiently concentrated in the bound fraction (Figure 5B).

The bound fractions collected from each of the NT and ERMO3-HA were then separated by SDS-PAGE and silver stained (Figure 5C). We found several proteins that were specifically located in the ERMO3-HA precipitate (indicated by asterisks). To comprehensively identify ERMO3/MVP1/GOLD36-binding proteins, whole proteins were subjected to MS analysis. The identified proteins are listed in Table S1. Seed-storage proteins contaminated the homogenate and were misidentified as ERMO3/MVP1/GOLD36-binding proteins (shown in oblique letters), since we used young seedlings that occasionally still contained seed coats. In addition to the comprehensive assay, several major proteins seen in the ERMO3-HA precipitate were excised from the stained gel and individually subjected to MS analysis. Excised bands and the identified protein names are indicated in Figure 5C and in Table S1 (bold oblique letters). Interestingly, most of the proteins identified with relatively high MASCOT scores were reported to be components of the PYK10 complex [26]. Identified proteins that are known to comprise the PYK10 complex, along with their homologues, are listed in Table 1. Among these proteins, interactions with either PYK10 or PBP1 were confirmed by immunoblot analysis (Figure 5D). PYK10 is the primary component of ER bodies, as described above, and the formation of the PYK10 complex may play an important role in the ER body system. This led us to speculate that there is a relationship between ERMO3/MVP1/GOLD36 and ER bodies.

**Table 1:** ERMO3/MVP1/GOLD36 interacts with PYK10 complex components.

Assigned Name or Annotation‡	AGI number	Score	Predicted Localization	Fold Change (Col/*nai1*) [26]	Correlation Coefficient with *NAI1*.†
**GLL23**	At1g54010	922	Secretory	3.10	-
**ERMO3/MVP1/GOLD36**	At1g54030	856	Vacuole*	3.00	0.44
**GLL22**	At1g54000	737	Secretory	2.36	-
ESM1/GLL65	At3g14210	118	Secretory	-	−0.18
GLL24	At1g54020	49	Secretory	-	−0.03
**JAL34**	At3g16460	389	Cytosol	3.30	0.65
**PBP1/JAL30**	At3g16420	159	Cytosol*	6.31	-
**MATH-domain containing protein**	At1g58270	112	unknown	0.97	0.48
**MATH-domain containing protein**	At3g20370	43	unknown	-	0.65
**PYK10/BGLU23**	At3g09260	252	ER body*	4.31	0.59
BGLU19	At3g21370	43	ER body	-	−0.09
BGLU18	At1g52400	43	ER body	-	−0.18

‡ Proteins with expression levels that are thought to be regulated by NAI1 are indicated by bold characters.

* The localizations of these proteins have been experimentally confirmed or suggested [28], [29], [33], [41].

† Correlation coefficients of expression patterns with *NAI1* (ATTED-II, http://atted.jp/).

### *NAI1* expression is important for the abnormal development of ER-aggregates in *ermo3*

**Figure 6:**
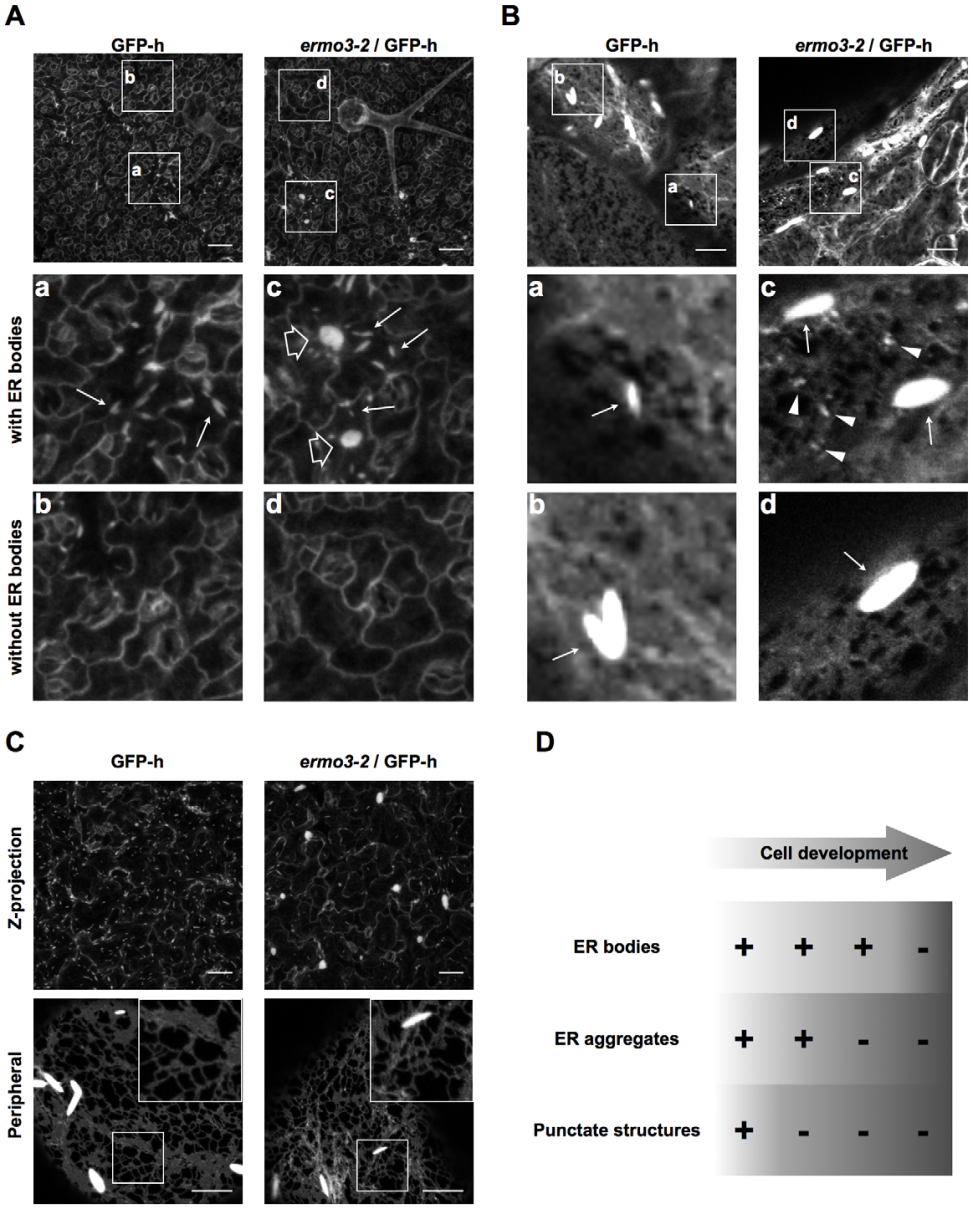
Development of ER bodies and ER-aggregates were highly correlated in *ermo3-1*. (**A**) and (**B**) Z-projected images (**A**) or confocal micrographs (**B**) of rosette leaves from 2-week-old GFP-h and *ermo3-2* plants. Enlarged views of boxed regions are shown in bottom panels (**a**) to (**d**). Arrows, open arrows, and arrowheads indicate ER bodies, ER-aggregate in *ermo3*, and aberrant punctate structures in *ermo3*, respectively. Bars, 50 µm (**A**) and 10 µm (**B**). (**C**) Z-projection (upper row; bars, 50 µm) or peripheral section (bottom row; bars, 10 µm) of cotyledon epidermal cells from 2-week-old GFP-h and *ermo3-2* plants. (**D**) Summarized phenotypes of *ermo3* cells during cell development.

Because *ERMO3/MVP1/GOLD36* was expressed ubiquitously (ATTED-II, http://atted.jp/; [28]; see Figure 3E), we expected that ER-aggregates would be evident in whole tissues of *ermo3* plants. Indeed, in 7-day-old seedlings, epidermal cells of petioles, hypocotyls, roots, and root hairs had ER-aggregates that were similar to those in cotyledonary cells (see Figure 1B). In contrast, surprisingly, most of the cells in rosette leaves from 2-week-old plants did not show any defects in ER morphology (Figure 6A, right column). Importantly, several cells from young rosette leaves still contained ER-aggregates (Figure 6Ac, open arrows). We found that these cells contained ER bodies as well (Figure 6Ac, arrows), while the other cells did not (Figure 6Ad). Observing peripheral punctate structures of ER body-developing cells, we noticed that some cells developed punctate structures and some did not (Figure 6B, right column). Furthermore, although the number was quite less, there were the cells with ER bodies but without ER-aggregates. To see the nature of punctate structures, we observed aged cotyledonary cells of 2-week-old plants and found that they had lost punctate structures from their ER network although they still develop the ER-aggregates (Figure 6C). These results suggest that the punctate structures were gradually suppressed during cell growth and the ER-aggregates were then also suppressed during further cell development (Figure 6D). Regardless of developmental stages of cells, our observation of rosette leaves at least indicates that the morphological defects in *ermo3* occurred where ER bodies were present.

ER body formation is regulated by a transcription factor named NAI1 [32] and, in other words, the cells that develop ER bodies are the cells where NAI1 expresses. Presence of NAI1 promotes expression of various proteins including PYK10, NAI2, and other ER body-related proteins. ERMO3/MVP1/GOLD36 is also one of the proteins that previously reported as NAI1-regulated proteins [26]. We tried to confirm this by qPCR (Figure S4A). In all tissues investigated of *nai1-1* mutant, ERMO3/MVP1/GOLD36 expression was partially reduced. The significant amount of remained expression indicates that regulation of its expression by NAI1 is limited, suggesting that ERMO3/MVP1/GOLD36 is expressed both in the cells with and without ER bodies. It further suggests that the cells with ER bodies required ERMO3/MVP1/GOLD36 for the proper organization of ER, while the cells without ER bodies were able to maintain ER morphology in the absence of ERMO3/MVP1/GOLD36 proteins.

**Figure 7:**
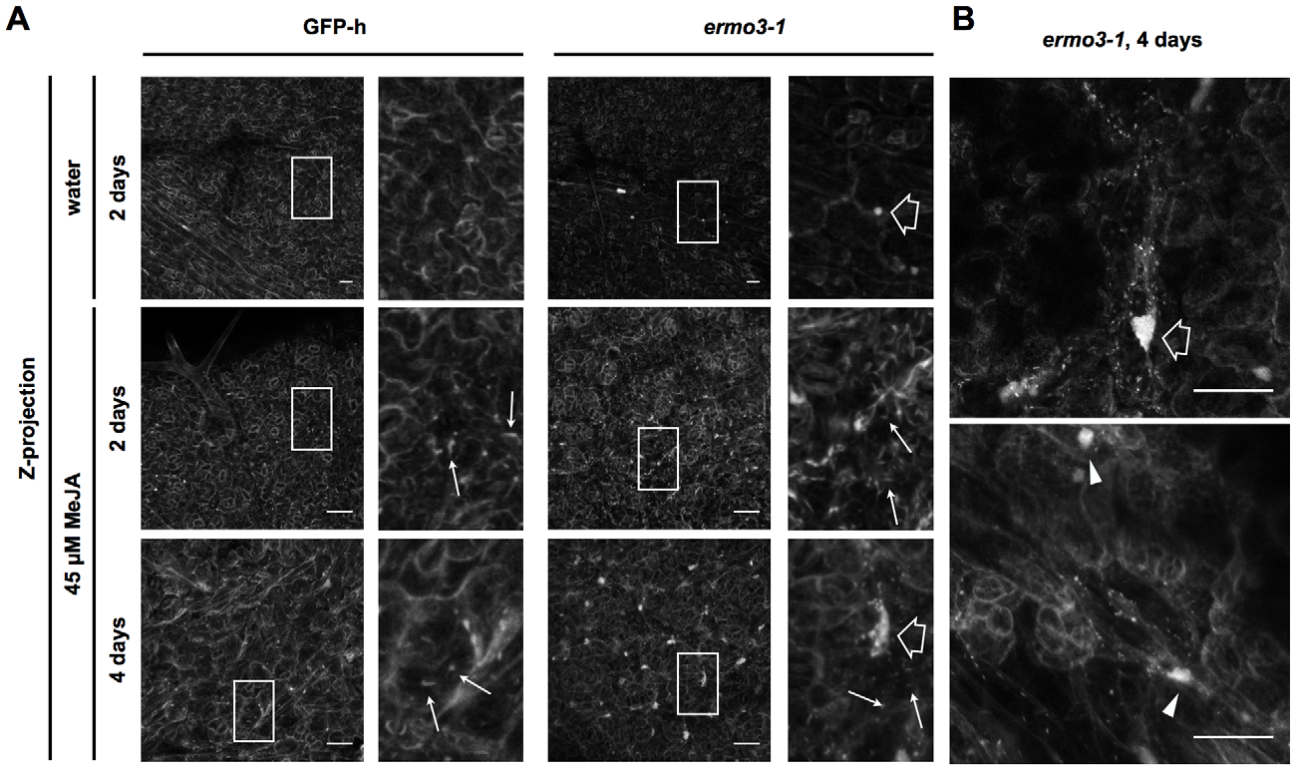
Induction of ER body formation by MeJA also induced ER-aggregate in *ermo3-1* rosette leaves. (**A**) Rosette leaves from 3-week-old plants were treated with 45 µM of methyl jasmonate (MeJA), or water as a solvent control, for the indicated number of days. 3D-reconstructed images are shown. MeJA treatment induced ER bodies in both GFP-h and *ermo3-1* cells (arrows). Notably, MeJA treatment co-induced ER-aggregate formation in *ermo3-1* cells (open arrows). (**B**) Higher magnification views of 4 day-treated *ermo3-1* cells are shown. Arrowheads indicate tangled ER, which might act as a “seed” of the ER-aggregates. Bars, 50 µm.

Since the biggest difference in molecular basis between these two types of cells was expression of NAI1, we then tried to examine the involvement of NAI1 in the aggregation of ER in *ermo3* cells. We performed two experiments: 1) the induction of ER body formation in *ermo3* rosette leaves by MeJA treatment, and 2) the depletion of NAI1 from *ermo3* cells. As mentioned above, NAI1 expression and ER body formation can be induced by MeJA treatment in rosette leaves, where ER bodies are absent under normal conditions. Rosette leaves from 3-week-old plants were collected and incubated in MeJA-containing water (Figure 7). After 2–4 days of incubation, ER bodies were clearly induced in GFP-h rosette leaves (Figure 7A, GFP-h, indicated by arrows). ER bodies were also induced in 4-day-incubated *ermo3-1* rosette leaves and, notably, large aggregates of ER were co-induced (Figure 7A, *ermo3-1*, indicated by open arrows), suggesting that there is a correlation between the formation of inducible ER bodies and ER-aggregates. In these cells, we often found tangled ER structures, which may act as “seeds” for ER-aggregation (Figure 7B, arrowheads). Since ER bodies that are on the way of formation are very small in size, we could not distinguish them from the ER-derived punctate structures.

**Figure 8:**
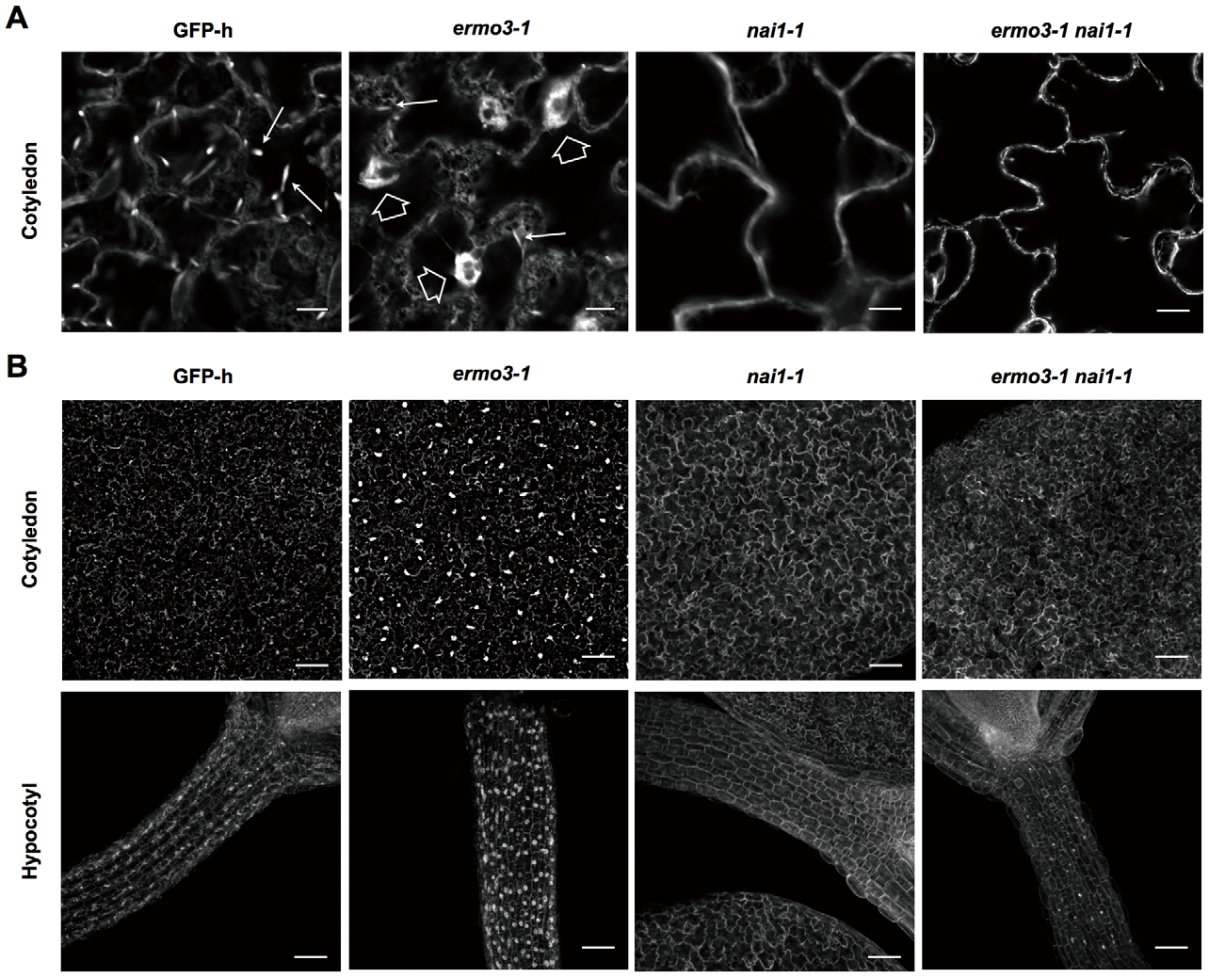
NAI1 expression was required for *ermo3-1* cells to form ER-aggregates. Depletion of *NAI1* in *ermo3-1* suppressed the formation of ER-aggregates. (**A**) Single optical sections of cotyledonary epidermal cells of indicated genotypes. Solid and open arrows indicate ER bodies and ER-aggregates, respectively. Bars, 10 µm. (**B**) 3D reconstructed images of cotyledon and hypocotyl. Bars, 100 µm.

Because MeJA also activates the expression of various responsive genes as well as *NAI1*, we then tried to determine the precise role of NAI1 by depleting NAI1 from *ermo3* cells (Figure 8). As previously reported, the *nai1-1* cells developed neither ER bodies nor ER-aggregates (Figure 8, *nai1-1*; [33]). To deplete NAI1 from *ermo3* cells, we crossed *ermo3-1* with *nai1-1* and generated an *ermo3-1 nai1-1* double mutant. In *ermo3-1 nai1-1* cells, neither ER bodies nor obvious ER-aggregates were found, which was also the case with *nai1-1* cells (Figure 8, *ermo3-1 nai1-1*). This suppression was not observed when either NAI2 or PYK10 was depleted from *ermo3-1* (Figures S4C and D); the expression of both of these proteins is positively regulated by NAI1 (Figure S4B). Importantly, *nai2-1* and *ermo3-1 nai2-1* cells did not have ER bodies, while *ermo3-1 nai2-1* obviously developed huge ER-aggregates identical to those seen in *ermo3-1* single mutant cells.

From our results, it was revealed that ER aggregation in *ermo3* was correlated with NAI1 expression but not with the formation of ER bodies: If it correlated with ER body formation, depletion of NAI2 from *ermo3* cells should have resulted in suppression of the morphological defects of ER. Our overall results clearly show that NAI1 expression in *ermo3* cells led ER to be disorganized. Because NAI1 is a transcription factor, protein(s) expressed under the positive control of NAI1 might be crucial for ER disorganization in *ermo3* cells. ERMO3/MVP1/GOLD36 may suppress this function in wild-type cells. Combined with our Co-IP results, these proteins are suggested to be PYK10 complex components.

### ER-aggregation is the trigger of defective protein transport

**Figure 9:**
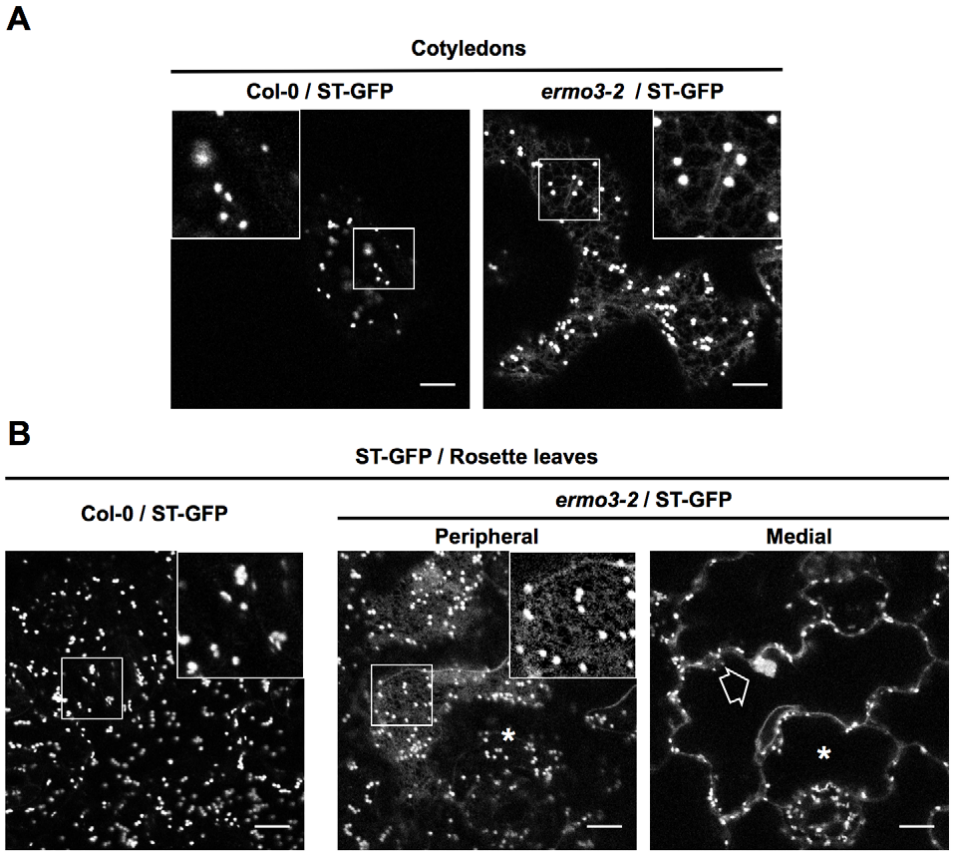
Defective protein transport was detected at the same place with where ER-aggregates were found. Cotyledons (**A**) and rosette leaves (**B**) from 10-day-old Col-0 and *ermo3-2* plants expressing ST-GFP were observed under a confocal microscope. In wild type cells, ST-GFP clearly labeled Golgi bodies. In *ermo3-2* cells, ST-GFP labeled ER network as well as Golgi bodies (insets). This labeling was detected in the cells with aggregates (an open arrow) but not in the cells without obvious aberrant structures (an asterisk). Bars, 10 µm.

According to two previous reports [28], [29], ERMO3/MVP1/GOLD36 might be involved in protein trafficking, as ST-GFP, secRFP, and GFP-PIP2a were mislocalized to the ER from the Golgi body, apoplast, and plasma membrane, respectively. To reveal if ERMO3/MVP1/GOLD36 was directly involved in protein transport, we fluorescently labeled Golgi bodies by crossing *ermo3-2* with ST-GFP-expressing Col-0 (Figure 9). ST-GFP is a membrane protein localized to *trans*-Golgi cesiternae and known to be dispersed to ER membrane when ER-Golgi protein transport is inhibited [15], [29]. In the cotyledonary epidermal cells of Col-0, ST-GFP clearly labeled Golgi bodies. In *ermo3-2* cells, as reported previously [29], ST-GFP labeled ER membranes as well as Golgi bodies, showing that ER-Golgi transport was inhibited in these cells (Figure 9A). Next we observed 2-week-old rosette leaves of Col-0 and *ermo3-2*. In wild type cells, as in cotyledonary cells, ST-GFP labeled Golgi bodies (Figure 9B, ST-GFP). In *ermo3-2* cells, ST-GFP clearly labeled ER network in the cell with aggregates (enlarged in inset), while it did not in the cell without aggregates (Figure 9B, *ermo3-2*, indicated by an asterisk). It indicates that the defects in ER-Golgi transport occurred only in the cells with the aggregates but not in the cells without aggregates. Combined with the suggestion that ERMO3/MVP1/GOLD36 expressed both types of the cells, our results suggest that ERMO3/MVP1/GOLD36 was involved in endomembrane organization itself, and the defective protein transport was due to disorganized ER-Golgi interface.

## Discussions

### ERMO3/MVP1/GOLD36 is required for maintaining ER morphology and organelle distribution

In this study, we isolated a mutant that has morphologically defective ER. We showed that ERMO3/MVP1/GOLD36 is required for maintaining ER morphology and organelle distribution (Figure 1). We found that only the ER body-developing cells contained ER-aggregates, while the rest of the cells in rosette leaves maintained proper ER morphology (Figure 6). Depletion of NAI1 from *ermo3-1* resulted in a total loss of ER-aggregates from whole-plant tissues (Figure 8). This suggests that a protein with expression that is positively regulated by NAI1 plays an important role in the formation of disorganized ER in *ermo3* cells. The leading candidate proteins, according to our Co-IP/MS analysis (Figure 5 and Table 1), are PYK10 complex components, most of which are expressed under the positive control of NAI1 [26], [32]; Table 1, right columns).

Unlike in the present study, ERMO3/MVP1/GOLD36 was previously shown not to interact with PYK10 but rather with TGG2 [28]. This discrepancy can be due to differences in experimental systems. We used seedlings to detect protein interactions in cells where ER-aggregates were seen, while the previous study employed rosette leaves, where TGG2 was abundant. PYK10 might not have been enriched enough to detect the interaction of ERMO3/MVP1/GOLD36 with PYK10 in the protein extract from rosette leaves. On the other hand, we did not detect a significant interaction between ERMO3/MVP1/GOLD36 and TGG2, perhaps because of the relatively low amount of TGG2 expression in seedlings. Thus, it is possible that ERMO3/MVP1/GOLD36 interacts with TGG2 in mature shoots and with PYK10 in roots and seedlings. Furthermore, we showed that the cells in rosette leaves, where ERMO3/MVP1/GOLD36 interacted with TGG2, did not contain endomembrane aggregates. It indicates that the interaction of ERMO3/MVP1/GOLD36 with PYK10 plays a role in maintaining ER morphology.

There are several mutants showing a defective distribution of organelles. For example, *katamari1* and *katmari2* were isolated by the aggregation of endomembrane marker SP-GFP-2SC [17], [34]. Also, *ermo2* showed aggregated organelles in addition to defective ER morphology [20], [21]. Among these mutants, *ermo2* and *kam1* developed somewhat similar aggregates, although the mechanisms by which the aggregates formed were different; *kam1* had defects in actin organization, while *ermo2* was defective in ER-Golgi protein transport and showed normal actin organization. The aggregate seen in an *ermo3* cell was not similar to those seen in any of the other mutants, suggesting that ERMO3/MVP1/GOLD36 is involved in another pathway. Furthermore, all mutants other than *ermo3* developed organelle aggregates in all tissues including rosette leaves, further suggesting a unique function for ERMO3/MVP1/GOLD36. It is very interesting that NAI1-regulated proteins utilize ubiquitously-expressed ERMO3/MVP1/GOLD36 in a specific type of cell to maintain the proper organization of organelles.

### ERMO3/MVP1/GOLD36 is localized to the vacuole but may function between the ER and Golgi bodies

Prior to this study, there were two reports with two different conclusions regarding the subcellular localization of ERMO3/MVP1/GOLD36 [28], [29]. Our results, that both ERMO3-tagRFP and ERMO3^G59S^-tagRFP labeled the vacuolar lumen (Figure S3), strongly support the vacuolar localization of ERMO3/MVP1/GOLD36. Despite of extensive studies by two distinct groups, the mechanism by which a vacuolar protein regulates ER morphology remained unclear. Here, we showed that ERMO3-Venus-HDEL complemented the morphological defects of the ER (Figure 4). Considering that native ERMO3/MVP1/GOLD36 was not sent back from Golgi bodies to the ER and that the mutated form of ERMO3/MVP1/GOLD36 with a defect of export from ER was not functional [29], our results suggest that export from the ER is necessary and sufficient for ERMO3/MVP1/GOLD36 to maintain ER morphology. Alternatively, it has been shown that part of the HDEL-bearing protein is transported to the vacuole and processed [33], [35]. Thus, although we could not see any obvious signals in the vacuole, it is possible that part of ERMO3-Venus-HDEL was transported to the vacuole and this transport was enough for ERMO3/MVP1/GOLD36 to suppress ER aggregation in *ermo3*-2 cells.

### ERMO3/MVP1/GOLD36 may be directly involved in organizing ER morphology but not in protein trafficking

Two previous reports suggested that ERMO3/MVP1/GOLD36 was involved in protein trafficking. The fact that secRFP, of which secretion was inhibited in *ermo3* cells [29], possesses a signal peptide but no specific transport signals suggests that ERMO3/MVP1/GOLD36 is required for general protein transport in *Arabidopsis thaliana*, at least in seedlings.

Our results suggest that the defective protein transport observed in *ermo3* cells was the secondary effect caused by abnormal organization of the ER. Then how did this disorganization occur? Interactions of ERMO3/MVP1/GOLD36 with PYK10 complex components lead us to hypothesize that ERMO3/MVP1/GOLD36 is involved in regulation of PYK10 complex. Because the PYK10 complex is a huge protein complex that engulfs various proteins, abnormal formation of the PYK10 complex in an intact cell can cause abnormal aggregation of ER and other organelles. ERMO3/MVP1/GOLD36 might function to inhibit formation of these complexes in intact cells. Protein export from the ER is mediated by COPII vesicles. Budding of COPII vesicles is launched by the activation of Sar1 GTPase by Sec12 followed by recruitment of the coat proteins, Sec23/24 and Sec13/31. These sequential events occur at specific sites in the ER called ER exit sites (ERES). Cargo proteins are captured by certain receptor proteins with cytosolic tails that interact with Sec24. As shown by transmission electron microscopy, the aggregates were crammed with membranous compartments and ER-derived membranes are closely attached homotypically (see Figure 1Cb, right column). It might inhibit proper organization of ERESs upon ER membranes and result in defective COPII formation.

### ERMO3/MVP1/GOLD36 and the ER body system

In this study, we clearly showed that ERMO3/MVP1/GOLD36 functions in a coordinated manner with NAI1. We also confirmed that ERMO3/MVP1/GOLD36 expression was partially depressed in *nai1-1* cells (Figure S4A), suggesting that ERMO3/MVP1/GOLD36 is involved in the ER body regulatory systems. Agee *et al.* proposed that ERMO3/MVP1/GOLD36 is required for ER body formation because the number of ER bodies was reduced in *mvp1-1* cells compared to wild-type cells. We could not, however, detect any obvious changes in the number of ER bodies in either *ermo3-1* or *ermo3*-2 cells. This might be due to the presence of ER bodies engulfed in the aggregates (see Figure 1Cb), which were slightly difficult to be counted. Moreover, we could not detect an interaction between ERMO3/MVP1/GOLD36 and NAI2, which is the most critical factor for ER body formation, leading us to conclude that the contribution of ERMO3/MVP1/GOLD36 to ER body formation is not highly significant.

ERMO3/MVP1/GOLD36 is a member of the GDSL-lipase/esterase family of proteins (GLLs). ERMO3/MVP1/GOLD36 and its closest homologues consist of a unique cluster that includes EPITHIOSPECIFIER MODIFIER1 (ESM1). ESM1 regulates glucosinolate breakdown by myrosinases and defines which compounds are produced when glucosinolates are hydrolyzed. In the presence of ESM1, isothiocyanates are predominantly produced, rather than simple nitriles [36]. A previous study suggested that there is a functional separation of GLL proteins based on the specificity of expression. ESM1 functions with TGG1 and TGG2 in leaves, GLL23 functions with PYK10 and/or other β-glucosidases in roots, and ERMO3/MVP1/GOLD36 functions in both places [26]. Indeed, ERMO3/MVP1/GOLD36 was shown to interact with TGG2 and possesses slight ESM1-like activity [28]. However, it is not yet clear how ERMO3/MVP1/GOLD36 functions in a similar manner to ESM1 without possessing any lipase/esterase activity. The relatively low effect of ERMO3/MVP1/GOLD36 depletion on the determination of hydrolysis products suggests another function for ERMO3/MVP1/GOLD36 rather than ESM1-like activity. Additionally, *esm1* did not display ER aggregation [28]. This further suggests that ERMO3/MVP1/GOLD36 might serve a different function from ESM1 and probably from other GLLs as well. It is possible that over the course of evolution, ERMO3/MVP1/GOLD36 has achieved additional functions that are required for proper trafficking of ER bodies/myrosinases-related proteins in both shoots and roots. It remains an interesting question whether ERMO3/MVP1/GOLD36 has ESM1-like activity toward various glucosinolates in roots. This might be difficult to investigate, however, because the isothiocyanates and nitriles that are derived from indole glucosinolates, which are abundant in *Arabidopsis* roots, are unstable. Still, however, it would be very interesting to investigate the functional differentiation among GLL proteins, since our results suggest that these proteins have evolved along a unique evolutionary pathway.

## Materials and Methods

### Plant materials and growth conditions

*Arabidopsis thaliana* (ecotype Columbia-0 and Landsberg *erecta*) was used as the wild type. Transgenic *Arabidopsis* GFP-h was generated as previously described [22]. Mutant lines *ermo3*-2 (SALK_135215) and *mvp1-2* (SALK_030621) were provided by the Arabidopsis Biological Resource Center (ABRC) at Ohio State University. Surface-sterilized seeds were sown onto MS medium containing either 0.5% Gellan Gum (Wako, Osaka, Japan) or 1% agarose supplemented with *myo*-inositol and 1% sucrose. Plants were grown at 22°C under continuous light. Isolation of *ermo3-1* and map-based cloning of *ERMO3/MVP1/GOLD36* were performed as previously described [20].

### Plasmid construction and transgenic plant generation

CDSs encoding ERMO3/MVP1/GOLD36 were amplified from a GFP-h cDNA library using gene-specific primers. Amplified products were subcloned into the pENTR/D-TOPO vector using a Gateway TOPO Cloning kit (Invitrogen). The G59S mutation was introduced into *ERMO3/MVP1/GOLD36* in pENTR/D-TOPO using site-directed mutagenesis. The subcloned DNA constructs were introduced into plant expression vectors (pGWB560 or pGWB514, donated from Dr. Nakagawa of Shimane University) for fusing with tagRFP or HA, respectively. To generate ERMO3-Venus-HDEL, we first cloned *SP-Venus-HDEL* into pENTR/D-TOPO. *SP-Venus-STOMAGEN* [37] was used as the template. HDEL was added by primer sequences. A Gly-Gly-Ala linker sequence containing *NcoI* sites was added to the 5′-end of ERMO3/MVP1/GOLD36 CDS and cloned into pENTR/D-TOPO. *NotI* and *NcoI* were used to combine ERMO3-GGA and Venus-HDEL. The resulting ERMO3-Venus-HDEL in pENTR/D-TOPO was introduced into pB2GW7. Primer sequences used in this study are shown in Table S2.

Each plant expression vector was introduced into *Arabidopsis* plants as previously described [20], [38]. Transformants were selected on plant growth media described above that contained the appropriate antibiotics. Protein expression of fluorescently-tagged constructs was confirmed under a fluorescence microscope. To confirm ERMO3-HA expression in seedlings, crude extracts from 7-day-old T2 plants (mixture of 20 individuals) of each transformant were subjected to immunoblot analysis, as described below.

### Fluorescence microscopy

The fluorescent images were obtained with a confocal laser scanning microscope (LSM780 META; Carl Zeiss) with either a 100×1.45 numerical aperture oil-immersion objective or a 40×0.95 numerical aperture dry objective. Images were analyzed with ZEN2010 software (Carl Zeiss) and processed with ImageJ (NIH).

### Electron microscopy

Seven-day-old seedlings of GFP-h and *ermo3-1* were fixed for 2 hours with 4% (w/v) paraformaldehyde and 1% (v/v) glutaraldehyde in 0.05 M cacodylate buffer, pH 7.4. Procedures for electron microscopy were essentially the same as those described previously [39]. The ultrathin sections were examined with a transmission electron microscope (model JEM-1011; JEOL) at 100 kV.

### RNA extraction and qPCR

RNA was isolated and cDNA was synthesized as previously described [20]. Primer sets used in conventional RT-PCR are presented in Table S2. Quantitative real-time PCR was performed with gene-specific primer sets (*ERMO3/MVP1/GOLD36*, At02212063_g1; *PYK10*, At02290764_gH; *NAI2*, At02252556_g1; *ACT2*, At02335270_gH; Applied Biosystems, http://www.appliedbiosystems.com/) and a TaqMan gene expression assay kit (Applied Biosystems) in a StepOnePlus Real-Time PCR System (Applied Biosystems). The relative quantity of the target cDNA was calculated using *Actin2* as a control. Statistical analysis was performed using R software (http://www.r-project.org). Parametric one-way ANOVA or Kruskal-Wallis's test was performed after Bartlett's test. Pair-wise comparisons were performed by Tukey's test.

### Coimmunoprecipitation

The coimmunoprecipitation assay was performed using a μMACS Epitope Tag Protein Isolation Kit (Miltenyi Biotec). Six-day-old seedlings (1 g) of non-transgenic Col-0 and ERMO3-HA were homogenized on ice in 4 mL of homogenization buffer (50 mM Tris-HCl [pH 7.5], 50 mM NaCl, 1% [v/v] Triton X-100, and Complete proteinase inhibitors [Roche, http://www.roche.com]). Cell debris was removed by centrifugation at 9,000 *g* for 20 min at 4°C and the resulting supernatants were collected as total fractions. Total fractions were incubated with 150 µL of magnetic beads conjugated to an anti-HA antibody (Milenyi Biotec) on ice for 30 min and then applied to μ columns (Miltenyi Biotec) in a magnetic field. The unbound flow-through fractions were also collected. After extensive washing with homogenization buffer, magnetic beads were eluted by, and boiled in, SDS sample buffer (100 mM Tris-HCl [pH 6.8], 4% [v/v] SDS, 20% [v/v] glycerol, 5% [v/v] 2-mercaptoethanol) at 99°C for 5 min. Magnetic beads were removed by centrifugation at 20,000 *g* for 5 min at room temperature, and the supernatants were collected as bound fractions.

### SDS-PAGE, silver staining, and immunoblot analysis

SDS-PAGE and immunoblot analysis were performed as previously described [39]. Antibodies used and dilution ratios were follows: anti-PYK10(IM) [33], 1∶10,000; anti-NAI2/deltaSP [40], 1∶2,000; anti-PBP1-C [41], 1∶10,000; anti-Thio, 1∶10,000; anti-HA(16B12) (Funakoshi, Japan), 1∶1,000; and anti-BiP [42], 1∶10,000. Silver staining was performed using a PlusOne Silver Staining Kit, Protein (GE Healthcare Bio-Sciences).

### Peptide preparation for tandem mass spectrometry analysis

Individual protein bands in silver-stained gel were excised following in-gel digestion. For comprehensive protein identification, bound fractions were separated on a 3 cm SDS gel. Gels were separated into three fractions and subjected to in-gel digestion. Identified proteins from each fraction were merged and shown as a single result. For in-gel digestion, each excised gel fraction was treated twice with 25 mM ammonium bicarbonate in 30% (v/v) acetonitrile for 10 min followed by 100% (v/v) acetonitrile for 15 min and then dried in a vacuum concentrator. The dried gel was treated with 0.01 mg/mL trypsin in 50 mM ammonium bicarbonate and incubated at 37°C for 16 hours. The digested peptides were recovered twice with 20 µL of 5% (v/v) formic acid in 50% (v/v) acetonitrile. The extracted peptides were combined and then evaporated to 10 µL in a vacuum concentrator.

### Mass spectrometric analysis and database search

Liquid chromatography–tandem mass spectrometry (MS/MS) analyses were performed using the LTQ-Orbitrap XL-HTC-PAL system. Trypsin digests were loaded onto the column (100 mm internal diameter, 15 cm length; L-Column, CERI) using a Paradigm MS4 HPLC pump (Michrom BioResources) and an HTC-PAL Autosampler (CTC Analytics) and were eluted by a gradient of 5 to 45% (v/v) acetonitrile in 0.1% (v/v) formic acid for 26 min. The eluted peptides were introduced directly into an LTQ-Orbitrap XL mass spectrometer (Thermo) with a flow rate of 200 nL/min and a spray voltage of 2.0 kV. The range of the MS scan was m/z 450 to 1500. The top three peaks were subjected to MS/MS analysis. MS/MS spectra were analyzed by the Mascot server (version 2.3) in house [43] (http://www.matrixscience.com/) and compared against proteins registered in TAIR8. The Mascot search parameters were set as follows: threshold of the ion score cutoff, 0.05; peptide tolerance, 10 ppm; MS/MS tolerance, 0.8 Da; and peptide charge, 2+ or 3+. The search was also set to allow one missed cleavage by trypsin, a carboxymethylation modification of Cys residues, and a variable oxidation modification of Met residues.

## Supporting Information

Figure S1 The aggregates in *ermo3-1* include various organelles.
(PDF)

Figure S2 *ERMO3* was located on At1g54030.
(PDF)

Figure S3 Substitution of Gly to Ser did not affect ERMO3/MVP1/GOLD36 functions.
(PDF)

Figure S4 Depletion of either of PYK10 or NAI2 from *ermo3-1* was not sufficient to suppress ER-aggregation.
(PDF)

Table S1 ERMO3/MVP1/GOLD36-interacting proteins identified by anti-HA pull-down and following mass spectrometry.
(PDF)

Table S2 Primer sequences used in this study.
(PDF)
