# Supplementary material for: ERMO3/MVP1/GOLD36 Is Involved in a Cell Type-Specific Mechanism for Maintaining ER Morphology in Arabidopsis thaliana
Source: PLoS One. 2012 Nov 14;7(11):e49103. doi: 10.1371/journal.pone.0049103 (PMC3498303; doi:10.1371/journal.pone.0049103)
Supplement: Figure S1 — The aggregates in ermo3-1 include various organelles. (PDF) [file pone.0049103.s001.pdf]

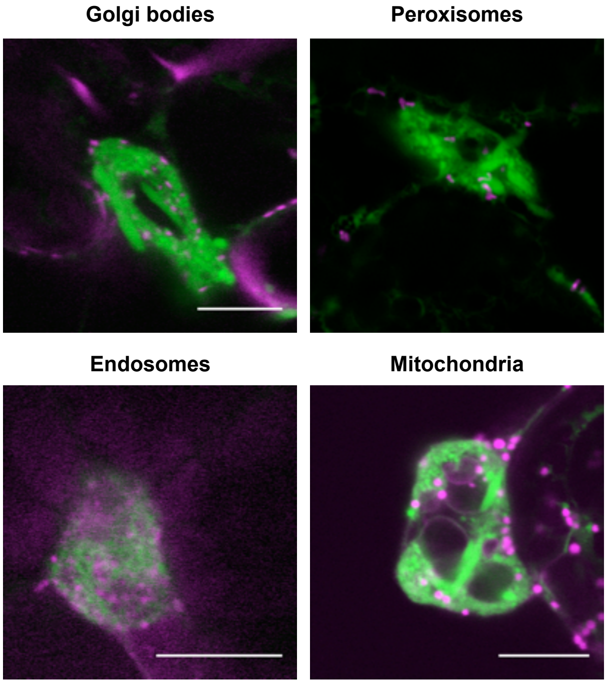

Supplemental Figure 1. The aggregates in *ermo3-1* include various organelles.
Golgi bodies, peroxisomes, and endosomes were labeled with the transient expression of RFP-fused marker proteins KAM1ΔC-mRFP, mRFP-PTS1, and Ara6-mRFP, respectively. Mitochondria were stained with MitoTracker Red CMRX. Bars, 10 μm.
