# Supplementary material for: ERMO3/MVP1/GOLD36 Is Involved in a Cell Type-Specific Mechanism for Maintaining ER Morphology in Arabidopsis thaliana
Source: PLoS One. 2012 Nov 14;7(11):e49103. doi: 10.1371/journal.pone.0049103 (PMC3498303; doi:10.1371/journal.pone.0049103)
Supplement: Figure S2 — ERMO3 was located on At1g54030. (PDF) [file pone.0049103.s002.pdf]

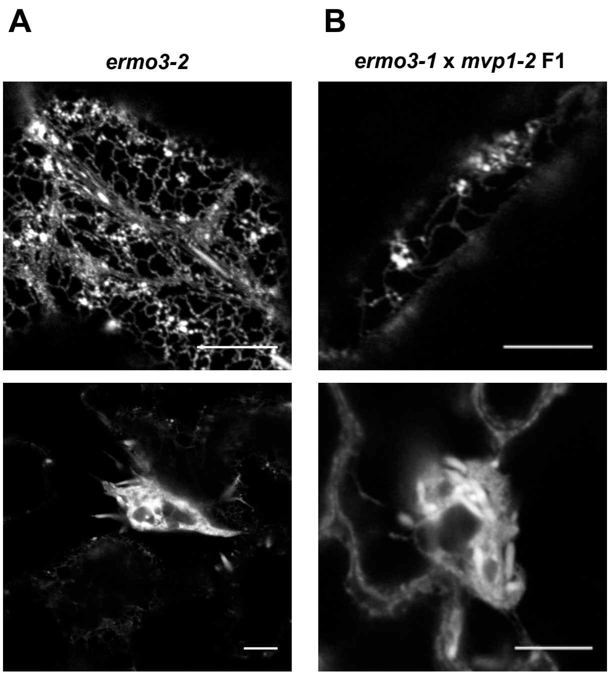

Supplemental Figure 2*.* *ERMO3* was located on At1g54030.
SP-GFP-HDEL was stably expressed in either *ermo3-2* (SALK_135215, **[a]**) or the F1 seedlings obtained from crossing *ermo3-1* and *mvp1-2* (SALK_030621, **[b]**). Cells of both genotypes developed punctate structures on the ER network (upper panels) and large aggregates of the ER (lower panels) that were indistinguishable from those in *ermo3-1*. Bars, 10 µm.
