# Supplementary material for: ERMO3/MVP1/GOLD36 Is Involved in a Cell Type-Specific Mechanism for Maintaining ER Morphology in Arabidopsis thaliana
Source: PLoS One. 2012 Nov 14;7(11):e49103. doi: 10.1371/journal.pone.0049103 (PMC3498303; doi:10.1371/journal.pone.0049103)
Supplement: Figure S3 — Substitution of Gly to Ser did not affect ERMO3/MVP1/GOLD36 functions. (PDF) [file pone.0049103.s003.pdf]

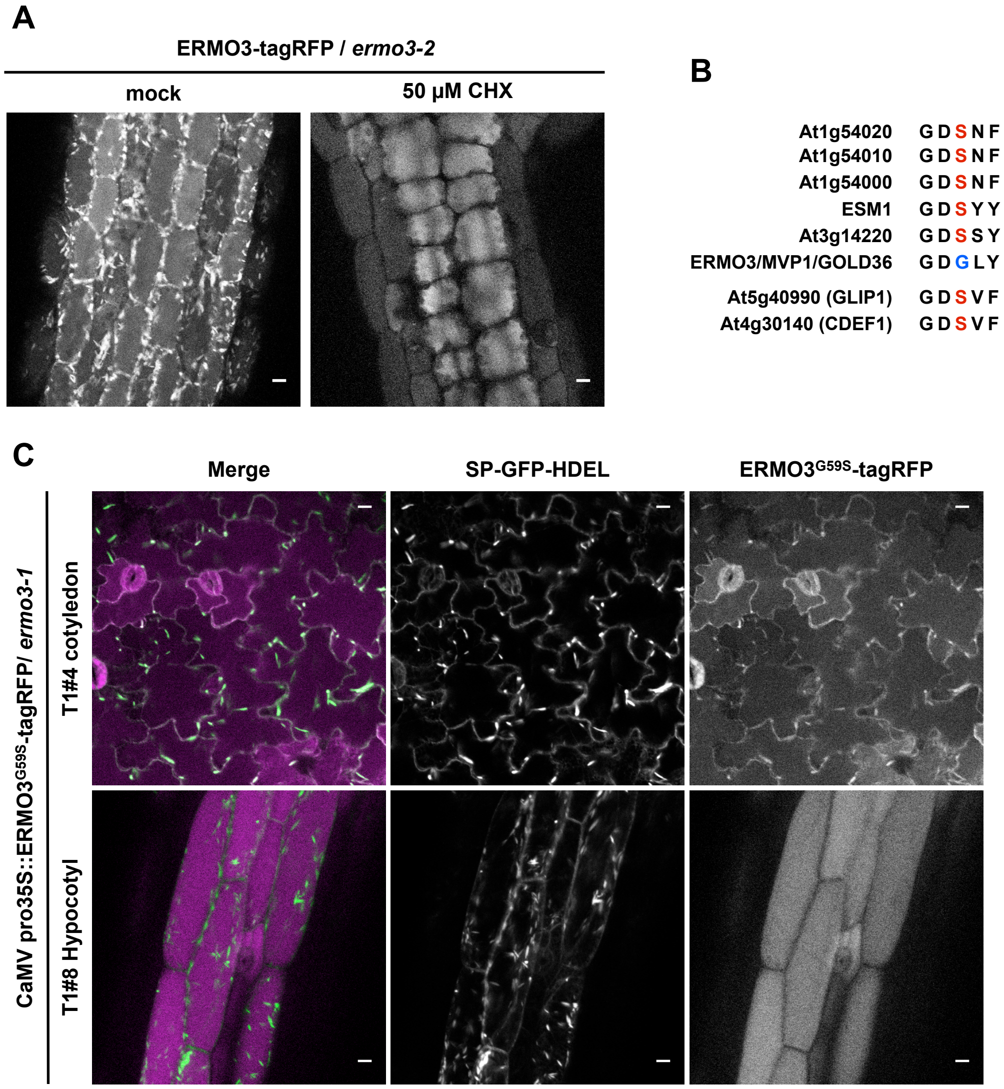

Supplemental Figure 3. Substitution of Gly to Ser did not affect ERMO3/MVP1/GOLD36 function. **(a)** ERMO3/MVP1/GOLD36 protein fused with tagRFP to its C-terminus was stably expressed in *ermo3-2*. The tagRFP signal was detected in the ER and vacuoles (left). A 50 µM cycloheximide treatment to inhibit protein synthesis completely excluded signals in the ER (right), indicating that ERMO3-tagRFP was transported from the ER to the vacuole. Note that no obvious aggregate was seen in the left panel, indicating the functionality of the fusion protein. Bars, 10 µm.
(b) Alignment of the GDSL motif in the catalytic center of ERMO3/MVP1/GOLD36 and homologues. Note that only ERMO3/MVP1/GOLD36 does not possess Ser residue (blue letter). This substitution was specifically seen in ERMO3/MVP1/GOLD36, among all other homologues (data not shown).
(c) ERMO3/MVP1/GOLD36 protein, with the proper catalytic center residue introduced (ERMO3^{G59S}), was fused with tagRFP and expressed in *ermo3-1*. T1 progeny were observed using a confocal microscope. ERMO3^{G59S}-tagRFP was properly transported to the vacuole, and SP-GFP-HDEL did not show any aggregates of ER, indicating that ERMO3^{G59S}-tagRFP was fully functional. Bars, 10 µm.
