# Supplementary material for: ERMO3/MVP1/GOLD36 Is Involved in a Cell Type-Specific Mechanism for Maintaining ER Morphology in Arabidopsis thaliana
Source: PLoS One. 2012 Nov 14;7(11):e49103. doi: 10.1371/journal.pone.0049103 (PMC3498303; doi:10.1371/journal.pone.0049103)
Supplement: Figure S4 — Depletion of either of PYK10 or NAI2 from ermo3-1 was not sufficient to suppress ER-aggregation. (PDF) [file pone.0049103.s004.pdf]

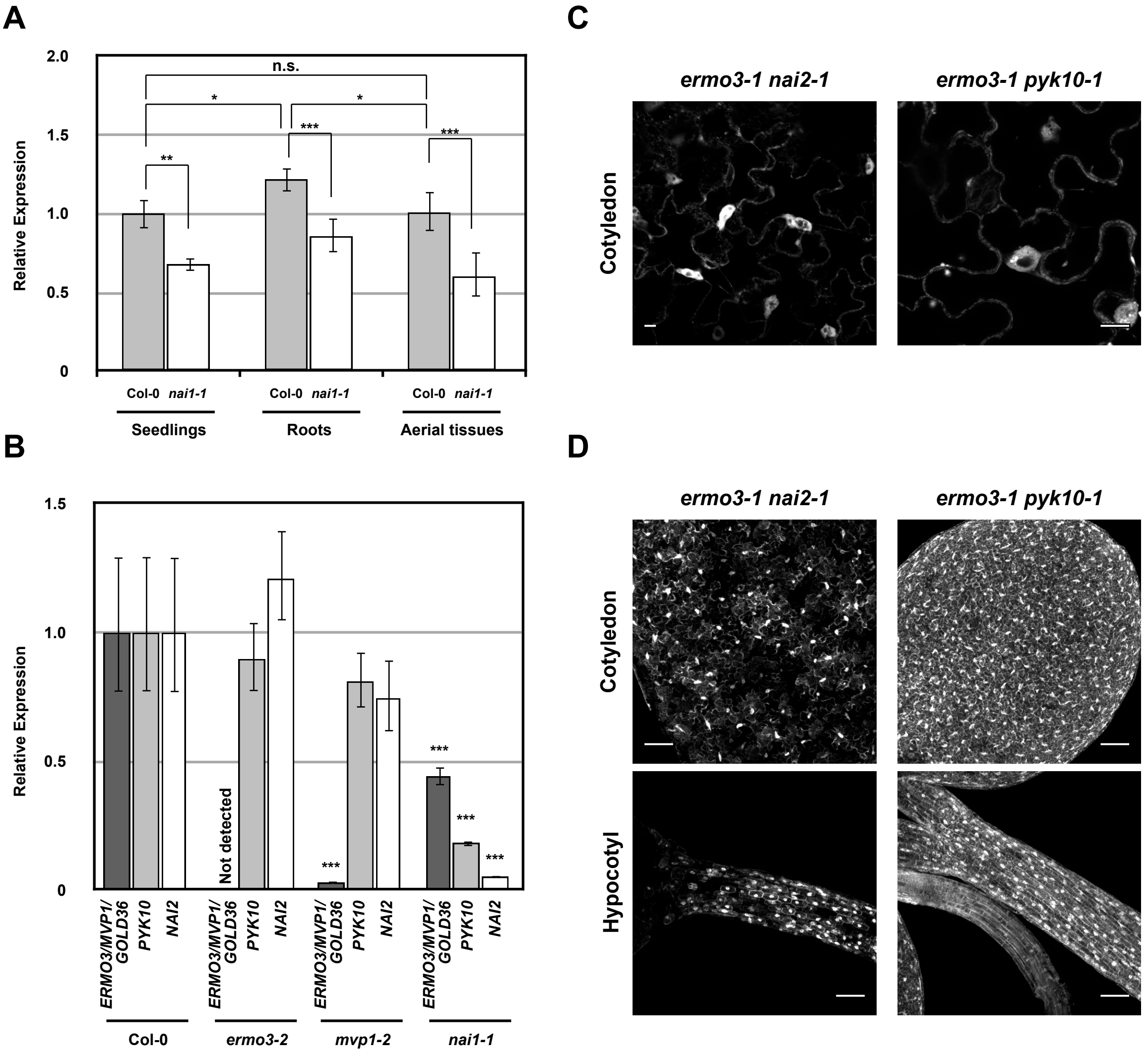

Supplemental Figure 4. Depletion of either of PYK10 or NAI2 from *ermo3-1* was not sufficient to suppress ER-aggregation.
(A) ERMO3/MVP1/GOLD36 expressed significantly less (* P<0.01, ** P<0.001, *** P< 0.0001) in *nai1-1* but still expressed.
(B) Expression of PYK10 and NAI2 were significantly reduced in *nai1* but not in *ermo3* (*** P< 0.0001).
(C) and **(D)** Confocal micrographs of double mutants generated by crossing *ermo3-1* with either *nai2-1* or *pyk10-1*. Images are shown as in Figure 8. Neither of these crosses suppressed the formation of ER-aggregates. Bars, 10 µm **(b)** and 100 µm **(c)**.
