# Supplementary material for: ERMO3/MVP1/GOLD36 Is Involved in a Cell Type-Specific Mechanism for Maintaining ER Morphology in Arabidopsis thaliana
Source: PLoS One. 2012 Nov 14;7(11):e49103. doi: 10.1371/journal.pone.0049103 (PMC3498303; doi:10.1371/journal.pone.0049103)
Supplement: Table S1 — ERMO3/MVP1/GOLD36-interacting proteins identified by anti-HA pull-down and following mass spectrometry. (PDF) [file pone.0049103.s005.pdf]

SCORE	AGI code	Gene name or annotations
922	**At1g54010**	**GLL23**
856	**At1g54030**	**ERMO3/MVP1/GOLD36**
737	**At1g54000**	**GLL22**
389	***At3g16460***	***JAL34***
375	*At4g28520*	*12S seed storage protein, putative / cruciferin, putative ((CRU3))*
345	*At5g50600*	*short-chain dehydrogenase/reductase (SDR) family protein*
252	***At3g09260***	***PYK10/BGLU23***
203	*At4g27160*	*2S seed storage protein 3 / 2S albumin storage protein / NWMU2-2S albumin 3*
199	*At4g27150*	*2S seed storage protein 2 / 2S albumin storage protein / NWMU2-2S albumin 2*
159	***At3g16420***	***PBP1***
118	**At3g14210**	**GLL65**
112	***At1g58270***	***ZW9; MATH-domain containing protein***
111	*At3g27660*	*glycine-rich protein / oleosin*
107	At1g20620	catalase 3 (SEN2) ((CAT3, SEN2))
103	*At3g01570*	*glycine-rich protein / oleosin*
87	*At4g27170*	*2S seed storage protein 4 / 2S albumin storage protein / NWMU2-2S albumin 4*
83	At3g26650	glyceraldehyde 3-phosphate dehydrogenase A, chloroplast (GAPA) / NADP-dependent glyceraldehydephosphate dehydrogenase subunit A ((GAPA))
78	At5g21274	calmodulin-6 (CAM6) ((CAM6))
72	At1g49240	actin 8 (ACT8) ((ACT8))
72	At1g42970	glyceraldehyde-3-phosphate dehydrogenase B, chloroplast (GAPB) / NADP-dependent glyceraldehydephosphate dehydrogenase subunit B ((GAPB))
65	At4g28390	ADP, ATP carrier protein, mitochondrial, putative / ADP/ATP translocase, putative / adenine nucleotide translocator, putative ((AAC3))
65	At5g13490	ADP, ATP carrier protein 2, mitochondrial / ADP/ATP translocase 2 / adenine nucleotide translocator 2 (ANT2) ((AAC2))
62	At1g17810	major intrinsic family protein / MIP family protein ((BETA-TIP))
61	At4g35090	catalase 2 ((CAT2))
56	At1g31340	ubiquitin family protein ((RUB1))
56	*At5g54740*	*protease inhibitor/seed storage/lipid transfer protein (LTP) family protein*
55	*At1g03880*	*12S seed storage protein (CRB) ((CRU2))*
49	**At1g54020**	**GLL24**
43	***At3g20370***	***MATH-domain containing protein***
43	***At3g21370***	***BGLU19***
43	***At1g52400***	***BGLU18***
43	*At5g44120*	*12S seed storage protein (CRA1) ((CRA1))*
39	At4g22010	multi-copper oxidase type I family protein
36	*At1g48990*	*glycine-rich protein / oleosin*
35	At5g58510	expressed protein
32	At1g80450	VQ motif-containing protein
30	At5g65950	expressed protein
29	At3g21000	expressed protein
28	At5g45520	hypothetical protein
28	At2g16640	chloroplast outer membrane protein, putative
28	At1g77280	protein kinase family protein
28	At4g15830	expressed protein
28	At5g23060	expressed protein
27	At2g40690	NAD-dependent glycerol-3-phosphate dehydrogenase family protein ((SFD1))
27	At5g02570	histone H2B, putative
27	At1g59980	DNAJ heat shock N-terminal domain-containing protein
27	At1g73360	homeobox-leucine zipper family protein / lipid-binding START domain-containing protein
27	At2g19240	RabGAP/TBC domain-containing protein
26	At3g63390	expressed protein
26	At1g73450	protein kinase, putative
26	At5g61160	transferase family protein
26	At3g01020	iron-sulfur cluster assembly complex protein, putative
26	At5g35980	protein kinase family protein
26	At2g13640	Golgi GDP mannose transporter (GONST1)
25	At3g44050	kinesin motor protein-related
25	At5g18820	chaperonin, putative
25	At4g31240	expressed protein
25	At3g19270	cytochrome P450 family protein ((CYP707A4))
24	At2g44410	expressed protein
24	At1g48920	nucleolin, putative
24	At2g37430	zinc finger (C2H2 type) family protein (ZAT11)
24	At1g19140	expressed protein
23	At3g13880	pentatricopeptide (PPR) repeat-containing protein
22	At5g14970	expressed protein
22	At5g18510	hypothetical protein
22	At5g16020	stress protein-related
21	At4g19230	cytochrome P450 family protein ((CYP707A1))
20	At5g20350	zinc finger (DHHC type) family protein / ankyrin repeat family protein
19	At5g20140	SOUL heme-binding family protein
19	At1g13610	expressed protein
19	At1g51480	disease resistance protein (CC-NBS-LRR class), putative
19	At1g06470	phosphate translocator-related
18	At3g06620	protein kinase family protein
17	At2g43760	molybdopterin biosynthesis MoaE family protein
16	At1g64670	hydrolase, alpha/beta fold family protein
14	At4g15210	beta-amylase (BMY1) / 1,4-alpha-D-glucan maltohydrolase ((ATBETA-AMY, RAM1))

Supplemental Table 1*.* ERMO3/MVP1/GOLD36-interacting proteins identified by anti-HA pull-down followed by mass spectrometry.
Proteins present in anti-HA precipitates from ERMO3-HA were identified by LTQ-Orbitrap mass spectrometry. Proteins also identified in anti-HA precipitates from non-transgenic CS60000 were excluded. Arabidopsis Genome Initiative (AGI) codes and annotations were obtained from the TAIR website (http://www.Arabidopsis.org/) and the ATTED-II website (http://atted.jp). Scores were calculated using Mascot software.
