# Supplementary material for: ERMO3/MVP1/GOLD36 Is Involved in a Cell Type-Specific Mechanism for Maintaining ER Morphology in Arabidopsis thaliana
Source: PLoS One. 2012 Nov 14;7(11):e49103. doi: 10.1371/journal.pone.0049103 (PMC3498303; doi:10.1371/journal.pone.0049103)
Supplement: Table S2 — Primer sequences used in this study. (PDF) [file pone.0049103.s006.pdf]

Primer name	Sequence (5’ - 3’)	Use
CACC-ERMO3-F	CACCATGCTTTTGATACCTTCCTTCACCGC	Subcloning of *ERMO3/MVP1/GOLD36*
ERMO3-R	TATCATAAAGGAGATAGCTTTGTCAACTTG	Subcloning of *ERMO3/MVP1/GOLD36*
ERMO3-GGA-NcoI-R	CCATGGCCCCCCCTATCATAAAGGAGATAG	Adding *Nco*I site to *ERMO3/MVP1/GOLD36*
ERMO3-G59S-F	CGGAGATAGTCTTTACGACGCCGGAA	Introduction of G59S mutation
ERMO3-G59S-R	TAAAGACTATCTCCGAACACAAAGAG	Introduction of G59S mutation
2S-SP-caccF	CACCATGGCCAGACTCACAAGCAT	Subcloning of *SP-Venus-HDEL*
Venus-HDEL-stop-R	TCAAAGCTCATCGTGCCCCCCCTTGTACAGCTCGTCCAT	Subcloning of *SP-Venus-HDEL*

Supplemental Table 2. Primer sequences used in this study.
